# Monitoring enteral feeding tolerance in a critically ill patient using point-of-care ultrasound

**DOI:** 10.1590/0034-7167-2025-0033

**Published:** 2025-12-08

**Authors:** Roberta Pereira Spala Neves, Allan Peixoto de Assis, Cesar Matias de Carvalho Russo

**Affiliations:** I Hospital Central Aristarcho Pessoa. Rio de Janeiro, Rio de Janeiro, Brazil

**Keywords:** Ultrasonography, Gastroparesis, Nutrition Therapy, Critical Care, Enteral Nutrition., Ultrassonografia, Gastroparesia, Terapia Nutricional, Cuidados Críticos, Nutrição Enteral., Ultrasonografía, Gastroparesia, Terapia Nutricional, Cuidados Críticos, Nutrición Enteral.

## Abstract

**Objectives:**

to describe the experience of using point-of-care ultrasound to monitor enteral nutrition tolerance in a critically ill patient.

**Methods:**

this is a nursing experience report on the use of bedside ultrasound to manage enteral feeding tolerance in an intensive care unit.

**Results:**

after unsuccessful attempts to optimize enteral nutrition, ultrasound-based monitoring of gastric volume revealed a significant delay in gastric emptying when gastric volume was compared to the administered feeding rate. This enabled early detection of gastroparesis and facilitated timely clinical decision-making for managing gastric intolerance.

**Final Considerations:**

this report highlights point-of-care ultrasound as a promising tool for monitoring enteral nutrition tolerance in critically ill patients and brings attention to the relevance of analyzing gastric volume in relation to the feeding rate.

## RESUMO

**Objetivos:**

relatar a experiência de uso do *point-of-care ultrasound* para acompanhamento da tolerância à terapia nutricional enteral de um paciente crítico.

**Métodos:**

trata-se de relato de experiência de enfermeiros no uso do ultrassom para manejo da tolerância à dieta enteral em uma unidade de terapia intensiva.

**Resultados:**

após fracassos na adequação da oferta nutricional enteral, iniciou-se o acompanhamento do volume gástrico utilizando o ultrassom e constatou-se que esse paciente apresentava atraso importante no esvaziamento gástrico quando comparado o volume gástrico à vazão da dieta ofertada. Isso possibilitou identificar precocemente a gastroparesia e acelerar a tomada de decisões para manejo da intolerância gástrica.

**Considerações Finais:**

este estudo aponta para o *point-of-care ultrasound* como um recurso promissor no acompanhamento da tolerância à dieta enteral em pacientes críticos e traz à luz a discussão sobre a análise do volume gástrico, relacionando-o à vazão da dieta oferecida.

## RESUMEN

**Objetivos:**

relatar la experiencia del uso del ultrasonido *point-of-care* para monitorear la tolerancia a la terapia nutricional enteral en un paciente crítico.

**Métodos:**

relato de experiencia de enfermeros que utilizaron el ultrasonido para gestionar la tolerancia a la nutrición enteral en una unidad de cuidados intensivos.

**Resultados:**

tras intentos fallidos de ajustar la oferta nutricional enteral, se inició el monitoreo del volumen gástrico mediante ultrasonido, identificándose un retraso significativo en el vaciamiento gástrico al comparar el volumen estimado con el flujo de la dieta. Este hallazgo permitió detectar precozmente la gastroparesia y agilizar las decisiones para el manejo de la intolerancia gástrica.

**Consideraciones Finales:**

el estudio señala el ultrasonido *point-of-care* como herramienta prometedora para el seguimiento de la tolerancia a la nutrición enteral en pacientes críticos y destaca la importancia de analizar dinámicamente el volumen gástrico en relación con el flujo administrado.

## INTRODUCTION

Nutrition support is a fundamental component of care for critically ill patients, with enteral nutrition being the first-line approach when the gastrointestinal tract is functioning normally. Critical illness triggers a cascade of metabolic and hormonal disturbances that may lead to varying degrees of inflammation, increased energy expenditure, and protein catabolism^(1)^.

Critically ill patients are predisposed to gastric dysmotility due to factors such as the use of hypokinetic medications, sepsis, surgical procedures, electrolyte imbalances, and acute illness^(1)^. When combined with enteral nutrition therapy, this condition increases the risk of aspiration and undernutrition.

Measuring gastric residual volume (GRV) through manual aspiration is no longer a routine practice and is typically indicated only in patients showing signs of feeding intolerance. However, there is no clear guidance on how to proceed based on the GRV values found^(2)^.

In the absence of intolerance signs, current recommendations advise suspending enteral feeding only when GRV exceeds 500 mL. For volumes between 200 mL and 500 mL, healthcare professionals should remain vigilant and implement strategies to reduce aspiration risk, such as administering prokinetic agents and following institutional protocols^(1,2)^. Studies have shown that estimating gastric volume by measuring the cross-sectional area of the gastric antrum (CSA) using point-of-care ultrasound (POCUS) can eliminate the need for manual aspiration^(3)^.

The term *point-of-care ultrasound (POCUS)* refers to the use of bedside ultrasound as an extension of the physical examination, supporting clinical decision-making and guiding procedures. Gastric ultrasound is a well-validated point-of-care application for assessing aspiration risk in patients undergoing emergency surgery who are unable to fast.

In this context, POCUS can also be used to estimate gastric volume in critically ill patients receiving enteral nutrition. Its use allows for early identification of feeding intolerance, guides strategies to minimize aspiration risk, and supports decisions regarding diet progression and the potential need for post-pyloric feeding tube placement.

## OBJECTIVES

To describe the experience of nurses using gastric point-of-care ultrasound (POCUS) to monitor enteral nutrition tolerance in a critically ill patient, emphasizing the advantages of this tool for early clinical decision-making, dynamic gastric volume assessment, and prevention of risks associated with gastric intolerance.

## METHODS

This is a nursing experience report on the use of gastric POCUS to manage enteral nutrition tolerance in an intensive care unit (ICU) at a military hospital in the state of Rio de Janeiro, Brazil, in 2023.

The patient’s legal representative authorized the use of clinical data, as well as ultrasound and radiologic images, for the preparation of this report. This authorization was documented through the signing of an Informed Consent Form (ICF), which was approved by the Research Ethics Committee.

## RESULTS

### Case presentation

A 66-year-old male patient with diabetes and chronic kidney disease was diagnosed with spondylodiscitis and admitted for antibiotic therapy. He was transferred to the intensive care unit (ICU) with sepsis. Ten days later, he developed septic shock and respiratory failure, requiring sedation and orotracheal intubation.

Beginning on ICU day 18, when the patient was already receiving his total energy requirement (TER), he began to show signs of feeding intolerance, including abdominal distension and emesis. Over the following week, the care team was unsuccessful in optimizing enteral nutrition delivery: each attempt to advance the feeding rate above 25 mL/h triggered signs of intolerance and elevated GRV, requiring the feeding to be suspended and then restarted.

**Figure 1:**
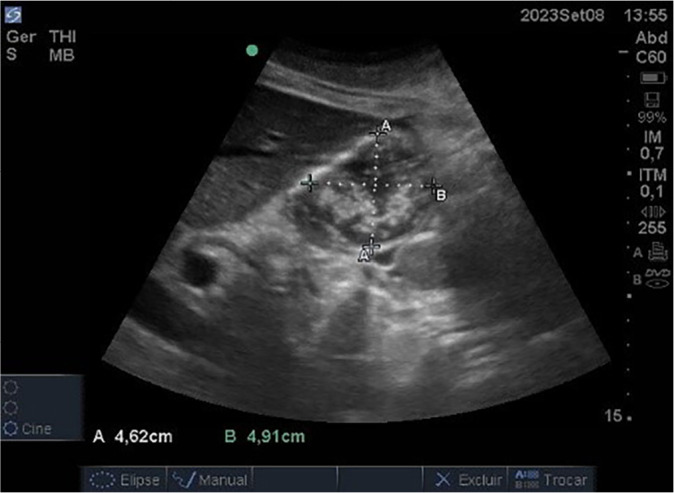
Gastric antrum ultrasound image on intensive care unit day 25

On ICU day 25, after a week of failed attempts to optimize nutritional support, a group of nurses initiated GV monitoring using point-of-care ultrasound (POCUS). An estimated GV of 202 mL was observed (Figure 1). Based on this finding, the team chose not to advance the feeding rate and initiated prokinetic therapy, despite the absence of overt signs of gastric intolerance. This decision was supported by an estimated gastric emptying (GE) delay of approximately eight hours, calculated from the ratio between GRV and the 25 mL/h infusion rate. On the following day, a repeat POCUS exam showed a GV of 193 mL, and the same management approach was maintained.

**Figure 2:**
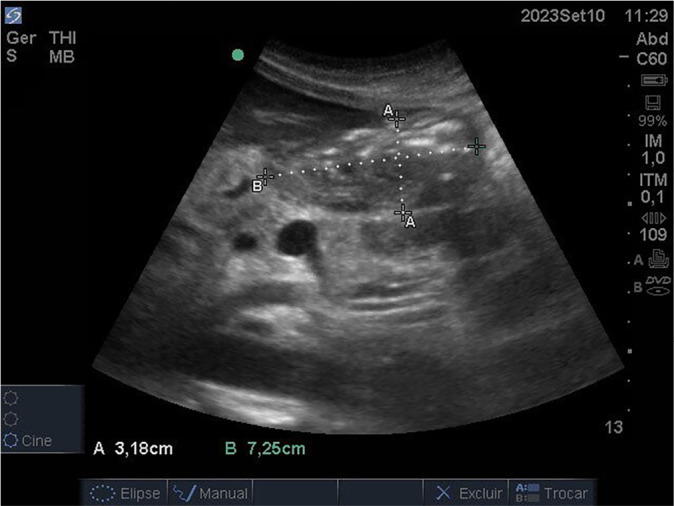
Gastric antrum ultrasound image on intensive care unit day 27

**Figure 3:**
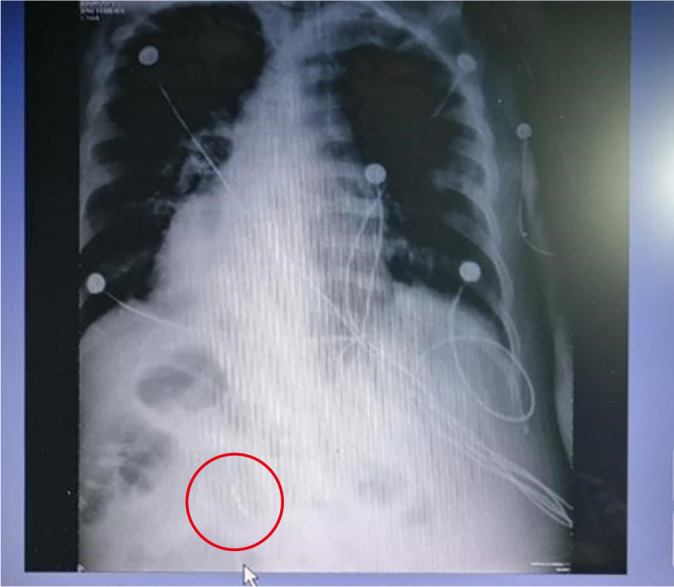
Radiograph following blind post-pyloric tube placement

On ICU day 27, the estimated GV was 206 mL (Figure 2), and the same infusion rate was maintained. The team decided to advance the feeding tube to a post-pyloric position. The nurse responsible for the patient performed the procedure blindly. A subsequent radiograph (Figure 3), reviewed and confirmed by the attending physician, verified the post-pyloric placement. The decision was made to maintain the current feeding rate for another 24 hours.

**Figure 4:**
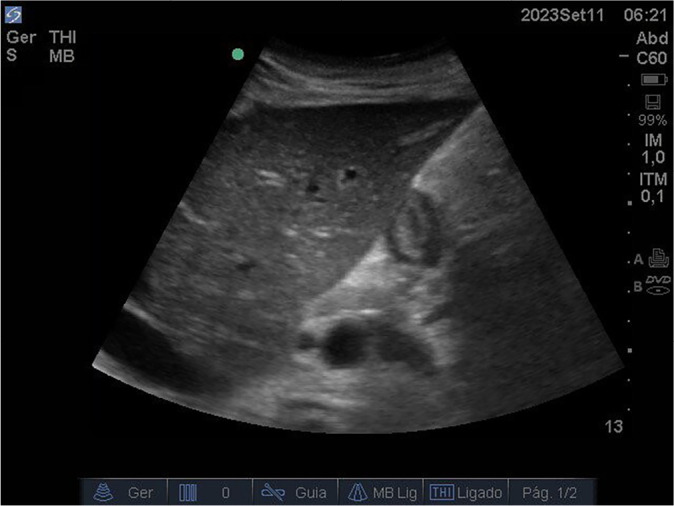
Gastric antrum ultrasound image on intensive care unit day 28

**Figure 5:**
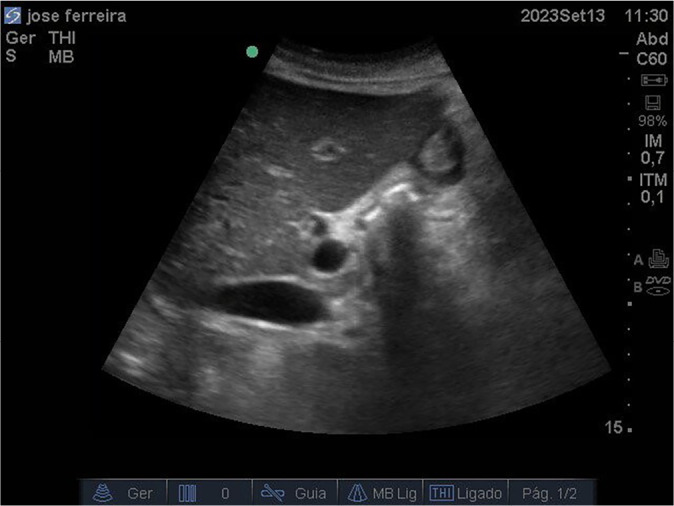
Gastric antrum ultrasound image on intensive care unit day 30

On the following day, another POCUS exam showed an empty stomach (Figure 4), allowing for progression of the feeding rate. POCUS exams continued for two additional days, confirming that the stomach remained empty (Figure 5). As a result, the patient’s TER was reached within three days.

## DISCUSSION

For many years, the routine measurement of gastric residual volume (GRV) was used to assess enteral feeding tolerance. However, according to the expert opinion reflected in the latest guidelines, its routine use is no longer recommended, as there is no consensus on a safe GRV threshold and this practice may compromise nutritional delivery and increase nursing workload^(2)^.

Another limitation of aspirating GRV to assess feeding tolerance relates to the distal positioning of the gastric tube. Depending on its location, it may not be possible to aspirate the entire gastric content, which can result in an underestimated GRV and significantly affect the accuracy of the feeding tolerance assessment^(4)^.

As a result, national and international guidelines now recommend monitoring enteral nutrition tolerance based on clinical signs, such as abdominal pain, distension, nausea, and diarrhea^(1,2)^. A range of complementary strategies should also be considered when monitoring critically ill patients receiving enteral nutrition therapy, including careful daily physical examination, radiographic confirmation of enteral tube placement, and evaluation of clinical risk factors for pulmonary aspiration^(1,2)^.

A study comparing aspirated GRV with gastric volume estimated through the ultrasound derived cross-sectional area of the antrum concluded that ultrasound allows accurate estimation of gastric volume in critically ill patients receiving enteral nutrition in the ICU. The authors also stated that nursing professionals can use bedside ultrasound as an alternative method for assessing gastric volume in patients with enteral feeding intolerance^(5)^.

A recent study demonstrated that nurses working in emergency, urgent care, and intensive care units recognize POCUS as a key tool in their clinical practice, as it significantly supports decision-making, enhances professional autonomy in performing procedures, and improves patient care delivery^(6)^.

It is also important to highlight that the Federal Nursing Council (Cofen), through Resolution No. 679/2021, authorizes nurses to perform bedside ultrasound in both hospital and pre-hospital settings to guide procedures and identify conditions that can be managed by nursing professionals^(7)^. Thus, POCUS has proven to be a promising resource for nurses in assessing enteral feeding tolerance and other aspects of nursing care, enabling the estimation of gastric volume and supporting the team in diagnosing gastric intolerance.

In this case, nurses applied gastric POCUS and performed a quantitative analysis of gastric volume by measuring the CSA using the technique described by Bolondi et al.^(8)^ and later by Perlas et al.^(9)^. The patient was placed in the right lateral decubitus position, and a convex transducer was positioned in the subxiphoid region, with the probe marker directed toward the patient’s head. The gastric antrum was visualized using the liver on the left and, beneath the antrum, the superior mesenteric artery and the aorta as anatomical landmarks. Two diameters were measured: craniocaudal (CC) and anteroposterior (AP). These values were then applied to the formula proposed by Bolondi et al.^(8)^ to calculate the cross-sectional area of the gastric antrum: ATAG = (CC × AP × π) /4.

Based on the CSA, GV was estimated using the following formula: GV = 27 + 14.6 × CSAGA (cm^2^) - 1.28 × age (in years)^(8,9)^.

Identifying the GV allowed for an assessment of the gastric emptying rate using the ratio between gastric volume and enteral feeding rate. For this analysis, we used a study that employed scintigraphy to compare GE in two groups (healthy individuals vs. critically ill patients) receiving enteral nutrition via gastric tube. The study concluded that GE is delayed when more than 13% of the administered volume remains in the stomach after 180 minutes and is significantly delayed when retention exceeds 82% at 240 minutes^(10)^.

In our case, the patient had an estimated GE delay of approximately 480 minutes. It was therefore concluded that advancing the feeding rate would likely result in an even higher GRV, potentially exceeding the 500 mL threshold set by current guidelines as the upper tolerance limit and increasing the risk of aspiration. This scenario had already been experienced by the team prior to using POCUS, when multiple attempts to advance feeding had failed.

The analysis of gastric emptying time-calculated using the ratio between the POCUS estimated GV and the feeding rate-guided clinical decisions to initially withhold feeding progression, prescribe a prokinetic agent, and place the enteral tube in a post-pyloric position. These measures promptly reduced the risks associated with feeding intolerance and enabled appropriate nutritional delivery.

### Study limitations

As this is a single-case experience report, the findings have limited generalizability, as the clinical response to assessing enteral feeding tolerance using POCUS observed in this patient may not reflect the broader behavior of critically ill patients with similar needs. Moreover, the proposed use of the ratio between gastric volume and feeding rate as a parameter to evaluate GE time-although innovative and effective in this case-still requires validation through more robust studies with appropriate methodology.

### Contributions to the field of nursing and healthcare

This study offers important contributions to nursing and healthcare practice, particularly in the context of intensive care and nutritional therapy. It presents an innovative approach to monitoring gastric tolerance to enteral nutrition using POCUS as a noninvasive assessment tool, enabling early detection of GE issues. This practice represents an advancement in nursing care for critically ill patients and aligns with Cofen Resolution 679/2021, which expands the nurse’s scope of practice in the use of bedside technologies.

In the broader healthcare context, the study enhances patient safety by reducing the risk of aspiration through early detection of gastroparesis, minimizing complications related to inadequate nutritional support, avoiding unnecessary invasive procedures such as repeated gastric aspiration, enabling continuous monitoring of gastric emptying, and facilitating the full delivery of planned caloric intake.

## FINAL CONSIDERATIONS

POCUS enabled the measurement of GRV, eliminating the need for manual aspiration and allowing for daily monitoring of gastric volume. By identifying gastric volume through POCUS, we could estimate gastric emptying time using the ratio between the GV and the feeding rate administered to the patient.

GE time proved to be a predictive factor for assessing feeding tolerance, facilitating clinical decision-making before the patient experienced adverse outcomes related to intolerance to enteral nutrition therapy.

This experience report highlights POCUS as a promising tool for monitoring enteral feeding tolerance in critically ill patients and brings attention to the importance of GV assessment, offering a dynamic perspective by relating it to the administered feeding rate.

However, further research is needed to explore the relationship between POCUS-based gastric emptying assessment and feeding tolerance in critically ill patients.

## Data Availability

The research data are available within the article.
